# Adsorption kinetics and mechanism of di‐*n*‐butyl phthalate by *Leuconostoc mesenteroides*

**DOI:** 10.1002/fsn3.1908

**Published:** 2020-10-12

**Authors:** Lili Zhao, Xinlei Li, Qingxiang Yang, Di Zhuang, Xin Pan, Lubo Li

**Affiliations:** ^1^ College of Life Sciences Henan Normal University Xinxiang Henan Province China; ^2^ Henan International Joint Laboratory of Agricultural Microbial Ecology and Technology Henan Normal University Xinxiang China

**Keywords:** di‐*n*‐butyl phthalate, *Leuconostoc mesenteroides*, removal mechanism

## Abstract

Di‐n‐butyl phthalate (DBP) poses a risk to humans as a ubiquitous environmental contaminant. A strain of *Leuconostoc mesenteroides* DM12 was chosen from lactic acid bacteria strains to study the DBP binding mechanisms. Adsorption of DBP by strain DM12 reached the highest binding rate of 87% after 11 hr of incubation, which could be explained by pseudo‐second‐order kinetics. The adsorption isotherm coincided with the model of Langmuir–Freundlich, indicating physical and chemical adsorption processes involved. Further, NaIO_4_ and TCA treatments were used to analyze the DBP binding mechanism of strain DM12, which indicated that peptidoglycan on the bacterial cell wall was involved in the process. The O‐H, C‐O, and N‐H bonds were possibly involved in the binding process as the main functional groups.

## Graphical abstract

The adsorption thermodynamics, kinetics and the binding sites of DBP by strain DM12.

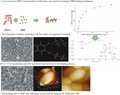

## 1 INTRODUCTION

Phthalates (PAEs) are a group of diesters of ortho‐phthalic acid, that is, dialkyl or alkyl aryl esters of 1, 2‐benzenedicarboxylic acid. PAEs have been monitored in many foods, food‐packaging materials, medical devices, cosmetics, and childcare equipment (Cao, 2010; Hernández‐Díaz et al., 2009; Silva et al., 2017). Exposure to PAEs occurs through water, food, air, soil, and dust by intake of chemicals via ingestion, inhalation, or adsorption through the skin (Fang et al., 2016). PAEs, as endocrine‐disrupting chemicals, can interfere with the action of human hormones (Nassouri et al., 2012). Di‐n‐butyl phthalate (DBP) is one type of PAEs, and it has been widely used as a solvent to retain color and scent in various consumer and personal care products, and in epoxy resins, cellulose esters, and special adhesive formulations (He et al., 2015). DBP is teratogenic in animals (Giribabu & Reddy, 2017; Silva et al., 2004). Bornehag et al. (2004) and Colón et al. (2000) demonstrated a relation between premature breast development, asthma, and allergic symptoms and DBP in children. Due to its toxicity, the European Food Safety Authority (EFSA) has specified the tolerable daily intake of DBP as 0.01 mg/kg body weight/day and China permits the maximum residue of DBP as 0.3 mg/kg in food and food additives. Therefore, DBP is a contaminant of major concern in the food industry, and there is an urgent need to establish an efficient method for its removal. However, biological removal of DBP in food, human, or other environments and its related mechanisms have not been reported.

Lactic acid bacteria (LABs) are a group of bacteria that can ferment lactose to lactate. Some strains of LABs have potential to reduce the risk of carcinogenic compounds and play a role in promoting health for humans (Clements & Carding, 2018; Sanders et al., 2014; Gibson et al., 2010). Some strains of LABs can lower genotoxicity and carcinogenic toxicity of heterocyclic aromatic amines via various components which can bind and remove these amines (Schwab et al., 2000). For example, *Enterococcus faecium* M74 and EF031 can bind aflatoxin B_1_ (AFB_1_) and patulin (Topcu et al., 2010). Zhu et al. (2017) reported that bisphenol A can be removed from aqueous solution by LAB strains via adsorption. Although previous studies showed that LAB strains are capable of removing cancerogenic compounds, the evidence about DBP binding by LAB strains is limited, and the removal mechanism and binding characteristics of carcinogenic compounds by bacterial cells need further research.

Previously, we discussed the adsorption of carcinogenic compounds, including polycyclic aromatic hydrocarbons, heterocyclic amines, and mycotoxins by LABs, and the various factors involve in this adsorption (Zhao et al., 2018). The aim of this study was to select an LAB strain to provide evidence to verify the binding characteristics and mechanism between bacterial cells and DBP. HPLC was used to investigate the DBP binding ability of bacterial cells. Scanning electron microscopy (*SEM*), atomic force microscopy (AFM), and Fourier transform infrared spectroscopy (FTIR) were used to ascertain the potential adsorption sites and investigate the possible binding mechanism of DBP by bacterial cells.

Modeling of adsorption isotherm results is considered essential for predicting and investigating adsorption mechanism (Chakravarty & Banerjee, 2012). The adsorption isotherms are reflected by adsorption properties and equilibrium data (Jeppua & Clement, 2012), describe how pollutants interact with adsorption materials (Crini et al., 2007). Therefore, they are critical in optimizing the use of adsorbents or the study of adsorption mechanism. In order to investigate the mechanism to remove DBP by LAB strains, it is important to find an appropriate correlation in the equilibrium curve. The common types of isotherms for analyzing experimental adsorption equilibrium data are the models of Langmuir, Freundlich, and the Langmuir–Freundlich (Azizian et al., 2007). This study characterized the DBP adsorption behavior of LABs with reference to the isotherm data analysis in terms of different models for better understanding of adsorption mechanism and explored the potential of the LAB strains in pollution control. We developed new probiotic functions of LAB strains to bind DBP and provided novel methods to remove PAEs from foods or human body.

## 2 MATERIALS AND METHODS

### 2.1 Chemicals

DBP standards were purchased from Lange Chemical Products Co. Ltd. (Beijing, China). Solvents for HPLC were of chromatographic grade (Thermo Fisher Scientific, Beijing, China). The LAB strains were cultured in de Mann Rogosa Sharpe (MRS) broth and agar (Oxoid, Basingstoke, UK). Other chemicals were obtained from Chemical Reagents Co. (Beijing, China).

### 2.2 LAB strains

LAB strains used in this study were isolated from various traditional fermented foods (*Lb. plantarum* CICC CICC 23121, CICC 22135, CICC 23138; *Lb. bulgaricus* CICC 6098; *B. longum* Y1, CICC 6186; *Lb. pentosus* CICC 22176, CICC 23156, CICC 23163; *Leuconostoc mesenteroides* DM12, CICC 23239, CICC 22731, CICC 22184; *Lb. brevis* CICC 6239; *B. breve* CICC 6079; *Lactococcus lactis* CICC 23195; *Lb. paralimentarius* CICC 22148), and stored in China Center of Industrial Culture Collection (CICC). In addition, *Lb. acidophilus* NCFM; *Bifidobacterium animalis* subsp*. lactis*, Hn019 and Bi04, and *Bifidobacterium bifidum* Bb02 were provided by Dupont, Shanghai.

### 2.3 Cell culture preparation

LAB strains were anaerobically cultured in sterile MRS medium, incubated at 37°C for 18 hr, and their cells were harvested by centrifugation at 5,000 ***g*** for 15 min at 4°C (Cenci et al., 2002). The pellets were washed twice with ultrapure water, and the cell concentration was determined using plate counting methods. Then, the pellets were suspended in sterile double‐distilled water and stored at 4°C before use.

### 2.4 DBP binding assay

#### 2.4.1 Binding rate

The DBP binding rates of LAB strains were calculated according to the method reported by Zhao et al. (2017). The binding assays include two steps: measure of DBP content of the solution by HPLC in positive (sterile H_2_O + DBP), negative (sterile H_2_O + cells) controls and samples (DBP + cells); and calculation of DBP binding rate of LAB strains using the equation:

(1) $$\text{Binding rate}\;\left(\%\right)=\left(1‐\frac{\text{DBP peak area of sample}}{\text{DBP peak area of positive control}}\right)\times 100$$

#### 2.4.2 Adsorption kinetics

The cultured LAB strain was centrifuged to obtain the bacteria. After washing with ultrapure water, the bacteria were suspended in a DBP solution (2–100 mg/L) to obtain a bacteria concentration of 1 g/L. The suspension was incubated with shaking at 37°C until adsorption equilibrium and then centrifuged at 5,000 ***g*** for 10 min to calculate the DBP equilibrium concentration of the system. Different adsorption isotherm models were used to fit the results.

Bacterial cells (0.001 g) were suspended in 1 ml DBP (50 mg/L) solution to a final concentration of 1 g/L. The mixed solution of DBP and bacterial cells was placed in a 37°C incubator and sampled every hour. The suspensions were centrifuged (5,000 ***g*** for 10 min at 4°C), and the supernatant was collected for analysis. The different adsorption kinetic models were used to fit the results.

The DBP adsorption of LAB strain was calculated using the equation:

(2) $$qe=\frac{(C0‐Ct)\times V}{m}$$

where *q_e_* is the DBP adsorption amount of LAB strain (mg/g), *C_o_* the initial concentration of DBP solution (mg/mL), *C_t_* the DBP concentration in the solution at time *t* (min), *V* the volume of the DBP solution (mL), and *m* the wet cell weight (g).

### 2.5 Binding stability of bacteria–DBP complex

To evaluate the stability of the bacterial cell–DBP complex, the complex was suspended in 1 ml of methanol or sterile water and incubated with shaking (37°C, 20 min). The bacterial cells were removed, and the supernatant was collected by centrifugation (5,000 ***g*** for 10 min at 4°C) and filtered with a 0.45‐μm filter. The DBP contents of supernatant and the percentage of DBP bound to bacterial cells were determined by HPLC analysis. All experiments were repeated three times.

### 2.6 Binding capacity of different cell components

The cell components that may be involved in the binding process were analyzed as the followings. The same number of cells (5.0 × 10^9^ CFU/mL) was used in following steps.

#### 2.6.1 Removal of exopolysaccharides from cell wall

One milliliter of 0.05 mol/L EDTA solution was added to the bacterial cells, and the suspensions were centrifuged (5,000 ***g***, 20 min) after an overnight shock to collect the bacterial precipitation of removal exopolysaccharides.

#### 2.6.2 Removal of surface protein from bacterial cell

One milliliter of 5 mol/L LiCl solution was added to the bacterial cells to obtain suspensions for reaction at 4°C for 60 min. The bacterial cells without surface protein were collected and washed twice with sterile water.

#### 2.6.3 Extraction of teichoic acid from cell wall

The bacterial cells were suspended in 10% TCA solution with shocking for 16 hr at 4°C. Then, the supernatant was collected and added with 2 volumes of cold ethanol after centrifugation (10,000 ***g***, 20 min). The mixture was further maintained at 4°C for 24 hr. Finally, the precipitate of teichoic acid (TA) was collected after centrifugation at 10,000 ***g*** for 20 min and washed in triplicate with sterile water.

#### 2.6.4 Extraction of peptidoglycan

One milliliter solution of 10% TCA was added to the bacterial pellets, incubated for 20 min in boiling water at 100°C, and quickly cooled to room temperature. The treated bacterial cells were collected by centrifugation (5,000 ***g***, 10 min), washed twice, and added with 2 volumes of mixture solution (chloroform: methanol 1:2, V: V), incubated at 4°C, 6 hr. After the above steps, the precipitate was collected by centrifugation (5,000 ***g***, 10 min), washed with deionized water for three times, and dissolved in Tris‐HCl buffer (0.1 mol/L, pH = 7.6). The buffer contained trypsin (3 mg/ml) and neutral protease (10 mg/ml). The bacterial cells were incubated in this buffer overnight (37°C, enzymatic hydrolysis) and centrifuged (10,000 ***g***, 20 min) to collect the intact peptidoglycan (PG) precipitation. The broken‐PG was obtained by disintegrating of intact PG via ultrasonic vibrations.

Finally, PG, broken‐PG, teichoic acids (TA), bacterial cells after removing exopolysaccharides (re‐EPS), and surface layer protein (re‐ S) were used to evaluate binding ability. The DBP binding rate was calculation as described by Zhao et al. (2017).

### 2.7 DBP binding site analysis

To verify the components of the bacterial cells that were mainly responsible for DBP adsorption, the cells of LAB strain were subjected to different chemical treatments to change the composition of the cells, and then the DBP binding rate of these treated cells was measured. The chemical agents used in these treatments were sodium periodate (NaIO_4_, 0.05 mol/L) and trichloroacetic acid (TCA, 0.6 mol/L) (El‐nezami et al., 2004; Haskard et al., 2000). The cells of test strains were suspended in NaIO_4_, cell concentration was adjusted to 5 × 10^9^ CFU/mL, and the reaction was carried out at 37°C for 12 hr. After the reaction, the pellets were washed with deionized water and used to determine the DBP adsorption rate. For TCA treatment, cells were suspended in TCA solution (5 × 10^9^ CFU/mL) and stirred in a boiling water bath for 20 min. The treated cells were quickly cooled to room temperature for DBP binding analysis.

### 2.8 Binding mechanism analysis

The analysis of *SEM*, AFM, and FTIR for chemically treated cells above was used to identify the potential functional groups and possible binding sites related to DBP binding. Firstly, *SEM* observation was performed by Hitachi S‐3400N II (Oriental Science and Technology Import and Export Co. Ltd., Beijing, China) to analyze the surface morphology and elementary compositions of chemically treated and untreated cells. Before analysis, all samples were freeze‐dried, coated with gold and fixed.

Secondly, the AFM images were obtained by using a Bruker (Multimode 8, Germany) microscope with a super‐high resolution scanner (scanning range in XY direction and Z direction ≤ 0.4 μm). The image acquisition was operated in tapping mode, room temperature, and the humidity of 50–60%. The automatic detection was carried in the smart needle entry mode using a Si_3_N_4_ probe, and the probe was mounted on a 200‐μm Olympus silicon cantilever, which had a 0.12 N/m of force spring constant.

Thirdly, to prepare for FTIR detection, KBr was added to the dry cells of LAB strain by the proportion of 1:100 and then ground to a powder and compressed into tablet form finally. The spectrum range was 4000–400 cm^−1^ by using a Bruker (Model Vertex 70) FTIR spectrometer. All the experiments were performed in triplicate.

### 2.9 Statistics

SPSS version 17.0 software was used for data analysis. Significant mean differences were calculated by least significant difference. Each experiment was conducted with three replicates.

## 3 RESULTS

### 3.1 Binding abilities of different strains

**Figure 1:**
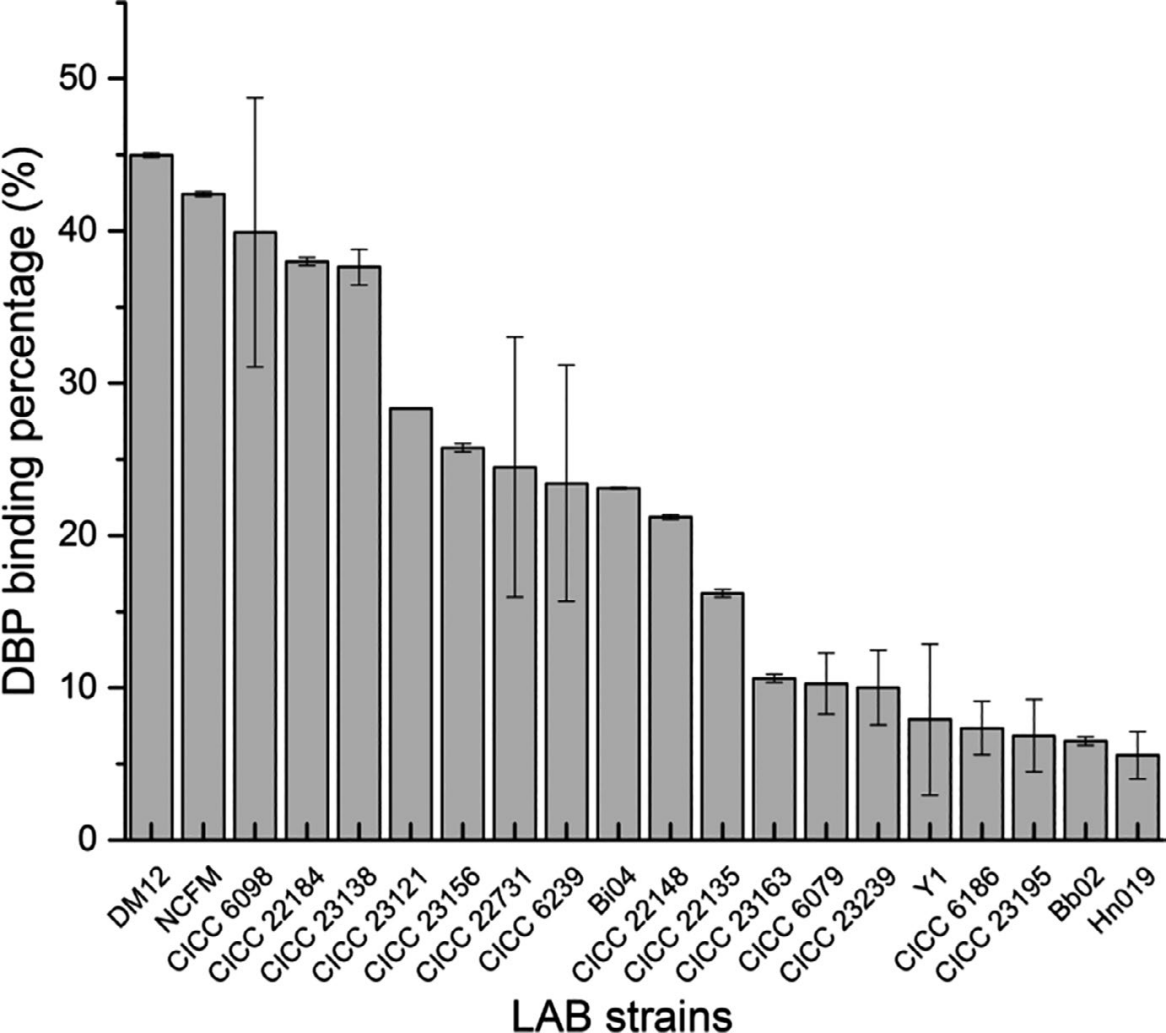
The binding abilities of different lactic acid bacteria strains

The 21 strains of LAB were tested, and the DBP binding ability was strain‐dependent with binding rate ranging from 5.48% to 44.98% (Figure 1). The DBP binding rate of *Leuconostoc mesogen* DM12 was better than that of other strains (*p* < .01). So, this strain was chosen for further study.

### 3.2 Adsorption kinetics and stability of DBP adsorption by strain DM12

The bacterial cells of strain DM12 were suspended in DBP solutions and sampled at different time points for DBP binding analysis. These results were fitted by different adsorption thermodynamic models to explain the thermodynamic properties and adsorption kinetics of strain DM12.

#### 3.2.1 Langmuir–Freundlich model

(3) $$qe=\frac{k\times C_{\mathrm{eq}}^{b}}{1+aC_{\mathrm{eq}}^{b}}$$

In the Equation 3, *q_e_* was the adsorption level of the bacteria in equilibrium, *k* and *a* the adsorption equilibrium constants, *b* an uneven parameter, *b* = 1 indicated that the surface of the absorbent surface was uniform, and *b* > 1 or *b* < 1 indicated uneven surface of the adsorbent (Umpleby et al., 2001).

**Figure 2:**
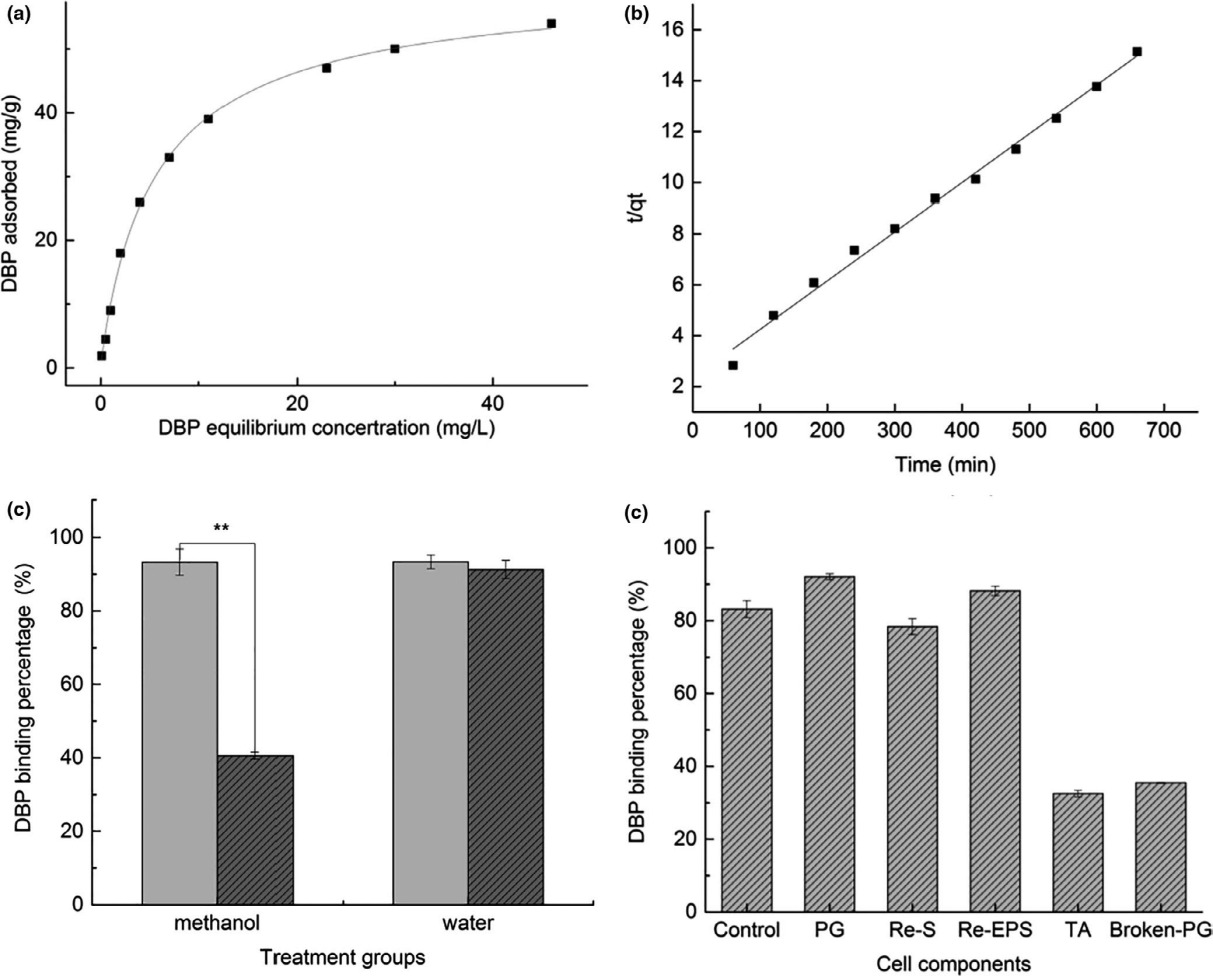
Adsorption kinetics, DBP binding stability of strain DM12, and the adsorption performance of various components of strain DM 12 cells

Unlike Langmuir or Freundlich models reflecting only the adsorption process of a single layer of molecules on a uniform surface, or the heterogeneous multilayer adsorption process, the kinetic form of Langmuir–Freundlich describes the adsorption kinetics of some basic concepts (like Langmuir model) and heterogeneity (Freundlich) in adsorption. That is, it reflects the heterogeneity of the surface energy of the adsorbent (bacteria) and the diversity of adsorption centers (Azizian et al., 2007). Therefore, it is more applicable to the complex component systems of bacterial cells, and predicting there was an interaction between DBP and the bacterial cells during physical and chemical adsorption (Chakravarty & Banerjee, 2012; Chatterjee et al., 2010). In this study, the relevant parameter characteristics of Figure 2a showed that Langmuir–Freundlich model had the better fit than Langmuir and Freundlich models to measure DBP adsorption of strain DM12, with an R^2^ (nonlinear regression coefficients of determination) value of 0.9981. So, Langmuir–Freundlich model better explained the DBP adsorption process of strain DM12. The parameter *b* = 0.9299 suggested that the adsorption process occurred on the surface of the uneven medium.

#### 3.2.2 Quasi‐secondary kinetic model analysis

The linear quasi‐secondary kinetic model was:

(4) $$\frac{t}{q_{t}}=\frac{1}{kq_{\mathrm{eq}}^{2}}+\frac{1}{q_{\mathrm{eq}}}t$$

*t* represented the time at which the adsorption reaction occurred; *k* was the quasi‐secondary adsorption rate constant; and *q_eq_* was the amount of bacterial adsorption at equilibrium (mg/g) (Ho, 2006).

The binding kinetics analysis showed that the adsorption amount increased continuously, which could be explained by pseudo‐second‐order kinetics equation (Figure 2b). The adsorption process was rapid at the beginning; the adsorption capacity was 21.17 and 25.00 mg/g DBP in the first and second hour, respectively. Then, the binding rate reached 87% after 11 hr of incubation, and the adsorption process was equilibrated gradually. The kinetics model of DBP adsorption by strain DM12 further illustrates that the adsorption process involves physical and chemical adsorption simultaneously (Yin et al., 2016).

The stability of DBP–bacterial complex was shown in Figure 2c. Fifty percent of DBP was washed off from the DBP–bacterial complex by methanol, but DBP adsorption remained stable after washing by water (*p* > .05). So, DBP bound to strain DM12 was easily washed off by methanol, and the physical adsorption played an important role in the binding process.

### 3.3 DBP binding ability of strain DM12 cell components

Strain DM12 was inactivated by heat treatment at 121°C, 15 min, then incubated with DBP at 37°C for 4 hr. The DBP adsorption rate did not change significantly after heat treatment (*p* > .05), indicating that the metabolic degradation was not involved in DBP removal.

DBP binding percentage of cell components (control, PG, TAs, Re‐ESP, and Re‐S) was determined by binding assay (Figure 2d), and the result showed that the highest binding percentage was PG (92.06%), followed by Removal of EPS (88.21%), which were higher than for complete cells (83.19%), indicating that EPS was not involved in the DBP binding process. The binding after removal of S‐layer protein showed a small decrease, which was not significant (*p* > .05). TA had an adsorption capacity of 32.53%. The DBP binding rate of PG in cell wall was highest among cell components. So, we will further focus on analyzing the DBP binding functional groups and binding sites of the cell wall PG.

### 3.4 Location of adsorption functional groups

**Figure 3:**
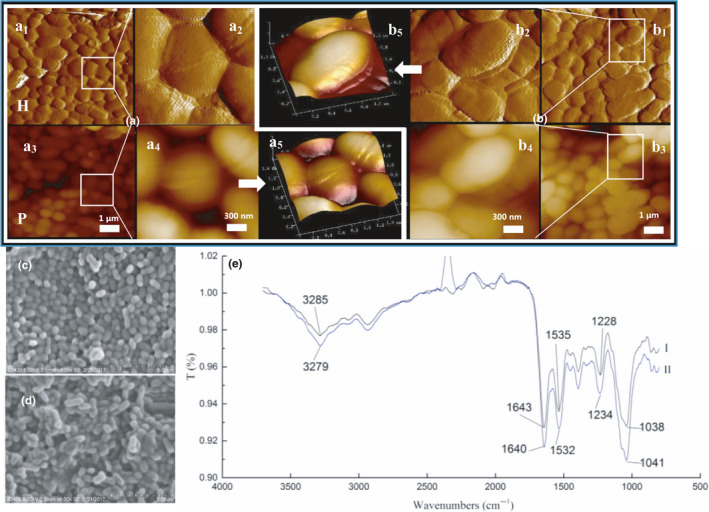
Locations of adsorption functional groups of DBP on the cell wall of strain DM12

Structural characteristics of the cell wall of strain DM12 were observed by *SEM* and AFM. *SEM* showed that strain DM12 cells had the unique morphology of *L. mesenteroides* with spherical cells appearing in pairs or clusters (Figure 3a,c). After binding to DBP, the surface of the bacterial cells began to be covered with a layer of oil‐like substances with a thickness of 20 nm. Moreover, the surface of the bacterial cells became smoother than the control group (Figure 3b,d). It was due to the adsorption of DBP to the bacterial cell wall surface.

FTIR showed that when DBP bound to strain DM12, the absorption peaks changed in the functional group and fingerprint areas. The main absorption bands were 370‐2800/cm and 1800‐800/cm (Figure 3e), and the absorption peaks were significantly stronger than those of bacteria without treatment. The peak at 3500‐3000/cm is the superposition of O‐H bonds stretching vibration of alcohol and phenol (Sun et al., 2009), indicating that the OH group of the bacteria was involved in DBP adsorption. The peak at 1650‐1400/cm is the stretching vibration of amide I, N‐H bending of amide II, and stretching vibration of amide III C‐*N* (Jain, 2003), whose strong characteristic absorption band and corresponding absorption peak displacement prove that proteins and peptides participate in DBP adsorption. The peak at 1228/cm is the stretching vibration of pyridine C‐N and polysaccharide P=O, and the wide peak at 1100–950/cm is generated by the stretching vibration of cell wall polysaccharide (Chen & Wang, 2008), indicating that bacterial polysaccharides participate in DBP adsorption. Therefore, it can be seen from the peak changes of infrared spectrum that the protein amide bond and the polysaccharide C‐O and O‐H bonds on the surface of the bacteria participate in the adsorption of DBP. So, the roles of protein and carbohydrate in the adsorption process were studied to find the binding sites in bacterial cells.

### 3.5 Binding sites of PG

**Figure 4:**
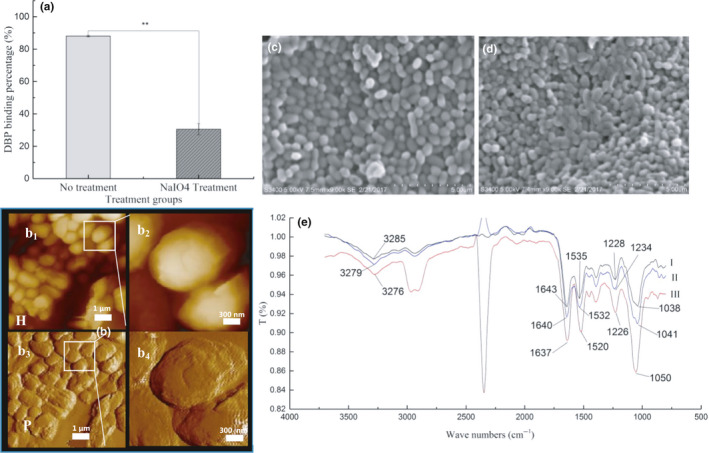
Binding ability of PG in the cell wall of strain DM 12 (NaIO4 treatment)

NaIO_4_ and TCA treatment were used to affect PG in the bacterial cell wall. After NaIO_4_ treatment, the DBP adsorption rate of strain DM12 was reduced significantly (57.5%, *p* < .01) (Figure 4a). *SEM* and AFM indicated that after treatment with NaIO_4_, the volume of cells was smaller than in the controls (strain DM12 without treatment), and their cell walls were partially broken (Figure 4b,d). These results suggested that NaIO_4_ treatment oxidized PG on the bacterial cells and damaged the cell wall structure, so the DBP binding rate of strain DM12 was decreased (Figure 4b,d).

FTIR showed that after NaIO_4_ treatment of strain DM12, the characteristic peak of DBP adsorption changed significantly compared with that of untreated bacteria (Figure 4e). The displacement of characteristic peaks was observed. The peak at 3279/cm moved to 3276/cm and the peak at 1041/cm moved to 1050/cm, indicating that the binding of hydroxyl and amino groups with DBP and the combination of C‐O bond and DBP of carbohydrate changed. The peak of amido III was moved from 1,234 to 1226/cm, and the displacement was decreased by 8 wave numbers. The peak of amide II moved from 1532 to 1520/cm, and the displacement was increased by 12 wave numbers. The peak of amide I shifted from 1,640 to 1637/cm. The results showed that the decreased DBP adsorption rate of strain DM12 after NaIO_4_ treatment might have been caused by changes in binding with carbohydrate O‐H and C‐O bonds, and protein amino N‐H and amide bonds, decreasing of number of binding sites and the interaction force.

**Figure 5:**
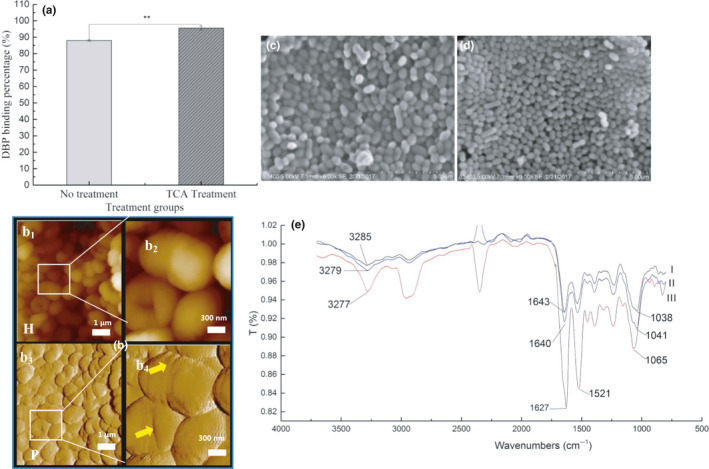
Binding ability of PG in the cell wall of strain DM 12 (TCA treatment)

After TCA treatment, DBP adsorption rate by strain DM12 increased (7.52%) (Figure 5a). *SEM* showed that TCA treatment reduced the volume of bacterial cells (Figure 5c,d). AFM showed that the TAs of strain DM12 on the cell wall surface were removed (Figure 5b), which might have exposed more adsorption sites on the PG, resulting in higher adsorption rate of strain DM12 cells.

FTIR showed that the absorption peak at 3279/cm moved to 3277/cm after TCA treatment (Figure 5e), and the absorption peak intensity was enhanced, indicating that the O‐H and amine N‐H of the cell wall increased and the number of hydrogen bonds formed increased. The absorption peak at 1640/cm was displaced to 1627/cm, indicating that the bond between amide I and DBP was strengthened. The peak at 1041/cm moved to 1065/cm, with an increase of 24 wave numbers, indicating that the binding sites of carbohydrate C‐O and DBP were increased. In conclusion, TCA treatment removed phosphotelic acid from the cell wall surface and increased the number of binding sites of PG with DBP on the cell surface of strain DM12, thus increasing the adsorption rate. In general, the functional groups mainly involved in DBP binding were hydroxyl, amino N‐H, amide, and C‐O bonds.

## 4 DISCUSSION

A large number of studies have confirmed that LAB strains can be an effective remover of food carcinogenic compounds, including polycyclic aromatic hydrocarbons, mycotoxins, and heterocyclic amines. *Lactobacillus* exerts anti‐cancer and anti‐mutation effects to prevent metabolism of carcinogenic compounds in the host (Zhao et al., 2013). Hernandez‐Mendoza et al. (2009) screened eight strains of *Lactobacillus casei* for adsorption of AFB1. AFB1 adsorption rates were strain specific and up to 49% Hatab et al. (2012) reported 10 strains of inactivated LABs that were used to adsorb patulin contaminants from apple juice and showed that *Lactobacillus rhamnosus* reduced the amount of PAT by 80.4%. In this study, *L. mesenteroides* DM12 screened from LAB strains was used to investigate DBP binding mechanism.

The adsorption process between adsorbent and adsorbate can be generally divided into physical and chemical adsorption (Crini et al., 2007; Yin et al., 2016). Physical adsorption is mainly achieved by intermolecular forces, no chemical bonds are formed and destroyed, electrons are not transferred, and atoms are not rearranged. Chemical adsorption is generally involving electronic sharing or electronic transfer (Hang et al., 2019). In order to study the mechanism of adsorption of DBP by strain DM12, the thermodynamics and kinetics of the adsorption process need to be investigated. To analyze further the interaction between the bacteria and DBP, the existing adsorption isotherm data were fitted with different models. The correlation coefficient *R* ^2^ of the three models showed that the Langmuir–Freundlich model had the best fitness (*R* ^2^ = 0.9981), which can describe the adsorption process of DBP by strain DM12. In the Langmuir–Freundlich adsorption model, b is a parameter of inhomogeneity. When *b* is 1, it means that the surface of the adsorbent is uniform, and the Langmuir–Freundlich equation becomes the Langmuir equation. The more the value of b deviates from 1, the more uneven is the surface of the adsorbent, reflecting the heterogeneity of the adsorbent surface and energy of the adsorption center. In this study, the adsorption of DBP by strain DM12 was consistent with the Langmuir–Freundlich model, which reflects the surface energy non‐uniformity of the adsorbent (bacteria) and the diversity of the adsorption center (Mishra, 2014), and the adsorption process may involve physical adsorption and chemical adsorption (Guo et al., 2014). The quasi‐second‐order kinetic equation is fitted to the experimental data, indicating that chemical adsorption may also participate in the adsorption process (Ho., 2006).

The binding stability of DBP bound to LAB strain is one of the important factors affecting their binding capacity (Zhao et al., 2013). Many researchers have evaluated the stability of LABs adsorbing carcinogens. Topcu et al. (2010) found that after PBS washing, *E. faecium* adsorption of AFB1 and PAT is still stable, with only 17%–23% of AFB_1_ and 19%–25% PAT being washed off. However, when bacterial cells were suspended in chloroform, 83%–99% AFB1 was washed away, probably because AFB1 compounds have similar polarity to chloroform (Haskard et al., 2001). In the present study, organic (methanol) and inorganic (water) solvents were used to measure the binding stability of strain DM12. DBP on bacterial cells was easily washed off by methanol, whereas binding was stable in water. This demonstrated that some LAB strains could be used to remove DBP from aqueous solutions.

Some studies have shown that the critical binding sites for the adsorption of patulin by *Lactobacillus* cell wall PG layers are N‐H, O‐H, and C‐O (Wang et al., 2015). Moreover, carbonyl oxygen C=O in AFB1 and acrylamide is attached to a hydroxyl group of a phosphoglycerol substituent or a glucose residue attached to a ribose chain (Serrano‐Niño et al., 2015). Zhang et al. (2017) showed that displacement of the C‐O bond (carboxyl group, polysaccharide, aromatics) and C=O and N‐H amide bonds was changed by infrared spectroscopy, which indicated that these three groups were involved in acrylamide binding. In this study, FTIR, *SEM*, and AFM were used to investigate the DBP binding sites. The DBP binding rate was decreased significantly by treatment with NaIO_4_. *SEM* and AFM showed that the surface morphology of the cells was changed greatly. The color of the bacterial cells changed to white, and the cells became smaller with serious cell wall damage. The main sites of action of NaIO_4_ are carbohydrates, oxidizing O‐H of carbohydrates to aldehydes and carbonic acid groups on the cell surface (El‐Nezami et al., 2004; Haskard et al., 2000, 2001). FTIR showed that NaIO_4_ treatment decreased the number of hydroxyl and C‐O bonds of carbohydrates in the cell wall and affected the structure of amino groups and amide bonds in polysaccharide peptides. Thus, the interaction force between DBP and binding sites in bacterial cell walls was reduced, leading to decreased adsorption rate of DBP. TCA treatment changed the size of bacterial cells and decreased their volume. AFM showed that the outer membrane was removed and the PG layer was exposed. It has been reported that TCA reduces the amount of TA on the surface of cells (Wicken & Knox, 1970). In the present study, we observed that TCA treatment affected the binding of DBP to the hydroxyl groups, amino groups, amide bonds, and lipid bonds. So, TCA treatment changed the cell wall structure and increased its binding ability. Therefore, the adsorption of DBP by bacterial cells possibly relies on several factors, including amide bonds, hydroxyl groups, and C‐O bonds.

In conclusion, we selected a single strain of *L. mesenteroides* DM12, from LABs with high adsorption of DBP and used it for further investigation of its mechanisms of adsorption. The results indicated that the adsorption process of DBP by strain DM12 coincided with the Langmuir–Freundlich model and involved physical and chemical adsorption. *SEM*, AFM, and FITR indicated that the O‐H and C‐O bonds of the saccharides, and the N‐H amide bonds on the cell wall were involved as the main functional adsorption groups. Further analysis using NaIO4 and TCA treatments on the cells suggested that PG on the cell wall surface was involved in the DBP binding process. However, the three‐dimensional structure of the bacterial cell wall seems critical for DBP binding, which should be further investigated. This is believed to be the first in‐depth investigation of the DBP adsorption mechanisms of LABs and will provide useful reference for the food and health industries.

## ETHICAL APPROVAL

This study does not involve any human or animal testing.

## INFORMED CONSENT

Written informed consent was obtained from all study participants.

## References

[fsn31908-bib-0001] Azizian, S. , Haerifar, M. , & Basiri‐Parsa, J. (2007). Extended geometric method: A simple approach to derive adsorption rate constants of Langmuir‐Freundlich kinetics. Chemosphere, 68, 2040–2046. 17408722 10.1016/j.chemosphere.2007.02.042

[fsn31908-bib-0002] Bornehag, C.‐G. , Sundell, J. , Weschler, C. J. , Sigsgaard, T. , Lundgren, B. , Hasselgren, M. , & Hägerhed‐Engman, L. (2004). The association between asthma and allergic symptoms in children and phthalates in house dust: a nested case‐control study. Environmental Health Perspectives, 112(14), 1393–1397. 15471731 10.1289/ehp.7187 PMC1247566

[fsn31908-bib-0003] Cao, X. L. (2010). Phthalate esters in foods: sources, occurrence, and analytical methods. Comprehensive Reviews in Food Science & Food Safety, 9(1), 21–43. 10.1111/j.1541-4337.2009.00093.x 33467808

[fsn31908-bib-0005] Cenci, G. , Rossi, J. , Trotta, F. , & Caldini, G. (2002). Lactic acid bacteria isolated from dairy products inhibit genotoxic effect of 4‐nitroquinoline‐1‐oxide in SOS‐chromotest. Systematic & Applied Microbiology, 25(4), 483–490. 12583707 10.1078/07232020260517607

[fsn31908-bib-0006] Chakravarty, R. , & Banerjee, P. C. (2012). Mechanism of cadmium binding on the cell wall of an acidophilic bacterium. Bioresource Technology, 108, 176–183. 22261660 10.1016/j.biortech.2011.12.100

[fsn31908-bib-0007] Chatterjee, S. , Das, S. K. , Chakravarty, R. , Chakrabarti, A. , Ghosh, S. , & Guha, A. K. (2010). Interaction of malathion, an organophosphorus pesticide with rhizopus oryzae biomass. Journal of Hazardous Materials, 174(1–3), 47–53. 10.1016/j.jhazmat.2009.09.014 19783095

[fsn31908-bib-0008] Chen, C. , & Wang, J. (2008). Removal of Pb^2+^, Ag^+^, Cs^+^, and Sr^2+^, from aqueous solution by brewery's waste biomass. Journal of Hazardous Materials, 151(1), 65–70. 17604909 10.1016/j.jhazmat.2007.05.046

[fsn31908-bib-0009] Clements, S. J. , & Carding, S. R. (2018). Diet, the intestinal microbiota, and immune health in aging. Critical Reviews in Food Science and Nutrition, 58(4), 651–661. 27712080 10.1080/10408398.2016.1211086

[fsn31908-bib-0010] Colón, I. , Caro, D. , Bourdony, C. J. , & Rosarlo, O. (2000). Identification of phthalate esters in the serum of young Puerto Rican girls with premature breast development. Environmental Health Perspectives, 108(9), 895–900. 10.1289/ehp.108-2556932 PMC2556932 11017896

[fsn31908-bib-0011] Crini, G. , Peindy, H. N. , Gimbert, F. , & Robert, C. (2007). Removal of C.I. Basic Green 4 (Malachite Green) from aqueous solutions by adsorption using cyclodextrin‐based adsorbent: Kinetic and equilibrium studies. Separation & Purification Technology, 53(1), 97–110.

[fsn31908-bib-0012] El‐Nezami, H. , Polychronaki, N. , Lee, Y. K. , Haskard, C. , Juvonen, R. , Salminen, S. , & Mykkänen, H. (2004). Chemical moieties and interactions involved in the binding of zearalenone to the surface of *Lactobacillus rhamnosus* strains GG. Journal of Agricultural and Food Chemistry, 52(14):4577–4581. 15237970 10.1021/jf049924m

[fsn31908-bib-0013] Fang, H. , Wang, J. , & Lynch, R. (2016). Migration of di (2‐ethylhexyl) phthalate (DEHP) and di‐n‐butylphthalate (DBP) from polypropylene food containers. Food Control, 73, 1298–1302.

[fsn31908-bib-0014] Gibson, G. R. , Scott, K. P. , Rastall, R. A. , Tuohy, K. M. , Hotchkiss, A. , Dubert‐Ferrandon, A. , Gareau, M. , Murphy, E. F. , Saulnier, D. , Loh, G. , Macfarlane, S. , Delzenne, N. , Ringel, Y. , Kozianowski, G. , Dickmann, R. , Lenoir‐Wijnkoop, I. , Walker, C. , & Buddington, R. (2010). Dietary prebiotics: Current status and new definition. Food Science & Technology Bulletin Functional Foods, 7(1), 1–19. 10.1616/1476-2137.15880

[fsn31908-bib-0015] Giribabu, N. , & Reddy, P. S. (2017). Protection of male reproductive toxicity in rats exposed to di‐n‐butyl phthalate during embryonic development by testosterone. Biomedicine & Pharmacotherapy, 87, 355–365. 28064108 10.1016/j.biopha.2016.12.106

[fsn31908-bib-0016] Guo, X. , Du, B. , Wei, Q. , Yang, J. , Hu, L. , Yan, L. , & Xu, W. (2014). Synthesis of amino functionalized magnetic graphenes composite material and its application to remove Cr (VI), Pb (II), Hg (II), Cd (II) and Ni (II) from contaminated water. Journal of Hazardous Materials, 278, 211–220. 25016452 10.1016/j.jhazmat.2014.05.075

[fsn31908-bib-0017] Hang, Y. , Si, Y. , Zhou, Q. , Yin, H. , Wang, A. , & Cao, A. (2019). Morphology‐controlled synthesis of calcium titanate particles and adsorption kinetics, isotherms, and thermodynamics of Cd (II), Pb (II), and Cu (II) cations. Journal of Hazardous Materials, 380, 120789 10.1016/j.jhazmat.2019.120789 31284171

[fsn31908-bib-0018] Haskard, C. , Binnion, C. , & Ahokas, J. (2000). Factors affecting the sequestration of aflatoxin by *Lactobacillus rhamnosus*, strain GG. Chemico‐Biological Interactions, 128(1), 39–49. 10996299 10.1016/s0009-2797(00)00186-1

[fsn31908-bib-0019] Haskard, C. A. , Elnezami, H. S. , Kankaanpää, P. E. , Salminen, S. , & Ahokas, J. T. (2001). Surface binding of aflatoxin B1 by lactic acid bacteria. Applied & Environmental Microbiology, 67(7), 3086–3091. 11425726 10.1128/AEM.67.7.3086-3091.2001 PMC92985

[fsn31908-bib-0020] Hatab, S. , Yue, T. , & Mohamed, O. (2012). Removal of patulin from apple juice using inactivated lactic acid bacteria. Journal of Applied Microbiology, 112(5), 892–899. 22394257 10.1111/j.1365-2672.2012.05279.x

[fsn31908-bib-0021] He, M. , Yang, C. , Geng, R. , Zhao, X. , Hong, L. , Piao, X. , Chen, T. , Quinto, M. , & Li, D. (2015). Monitoring of phthalates in foodstuffs using gas purge microsyringe extraction coupled with GC‐MS. Analytica Chimica Acta, 879, 63–68. 26002478 10.1016/j.aca.2015.02.066

[fsn31908-bib-0022] Hernández‐Díaz, S. , Mitchell, A. A. , Kelley, K. E. , Calafat, A. M. , & Hauser, R. (2009). Medications as a potential source of exposure to phthalates in the U.S. Population. Environmental Health Perspectives, 117(2), 185–189. 19270786 10.1289/ehp.11766 PMC2649218

[fsn31908-bib-0023] Hernandez‐Mendoza, A. , Guzmandepeña, D. , & Garcia, H. S. (2009). Key role of teichoic acids on aflatoxin B1 binding by probiotic bacteria. Journal of Applied Microbiology, 107(2), 395–403. 19486416 10.1111/j.1365-2672.2009.04217.x

[fsn31908-bib-0024] Ho, Y.‐S. (2006). Review of second‐order models for adsorption systems. Journal of Hazardous Materials, 136, 681–689. 10.1016/j.jhazmat.2005.12.043 16460877

[fsn31908-bib-0025] Jain, A. N. (2003). Surflex: Fully automatic flexible molecular docking using a molecular similarity‐based search engine. Journal of Medicinal Chemistry, 46(4), 499. 12570372 10.1021/jm020406h

[fsn31908-bib-0026] Jeppua, G. P. , & Clement, T. P. (2012). Modified Langmuir‐Freundlich isotherm model for simulating pH‐dependent adsorption effects. Journal of Contaminant Hydrology, 129–130, 46–53. 10.1016/j.jconhyd.2011.12.001 22261349

[fsn31908-bib-0028] Mishra, V. (2014). Biological removal of heavy metal zinc from industrial effluent by Zinc sequestering bacterium VMSDCM. Clean Technologies & Environmental Policy, 16(3), 555–568.

[fsn31908-bib-0029] Nassouri, A. S. , Archambeaud, F. , & Desailloud, R. (2012). Endocrine disruptors: Echoes of congress of Endocrinology in 2012. Annales D'endocrinologie, 73(8), 36–44. 10.1016/S0003-4266(12)70013-6 23089380

[fsn31908-bib-0030] Sanders, M. E. , Lenoir‐Wijnkoop, I. , Salminen, S. , Merenstein, D. J. , Gibson, G. R. , & Petschow, B. W. (2014). Probiotics and prebiotics: Prospects for public health and nutritional recommendations. Annals of the New York Academy of Sciences, 1309(1), 19–29. 24571254 10.1111/nyas.12377

[fsn31908-bib-0031] Schwab, C. E. , Huber, W. W. , Parzefall, W. , Hietsch, G. , Kassie, F. , & Schulte‐Hermann, R. (2000). Search for compounds that inhibit the genotoxic and carcinogenic effects of heterocyclic aromatic amines. Critical Reviews in Toxicology, 30(1), 1–69. 10680768 10.1080/10408440091159167

[fsn31908-bib-0032] Serrano‐Niño, J. C. , Cavazos‐Garduño, A. , Cantú‐Cornelio, F. , González‐Córdova, A. F. , Vallejo‐Cordoba, B. , Hernandez‐Mendoza, A. , & Garcia, H. S. (2015). In vitro, reduced availability of aflatoxin B1, and acrylamide by bonding interactions with teichoic acids from *lactobacillus* strains. LWT – Food Science and Technology, 64(2), 1334–1341.

[fsn31908-bib-0033] Silva, M. J. , Barr, D. B. , Reidy, J. A. , Malek, N. A. , Hodge, C. C. , Caudill, S. P. , & Brock, J. W. , Needham, L. L. , & Calafat, A. M. (2004). Urinary levels of seven phthalate metabolites in the U.S. population from the National Health and Nutrition Examination Survey (NHANES) 1999–2000. Environment Health Perspectives, 112(3), 331–338. 10.1289/ehp.6723 PMC1241863 14998749

[fsn31908-bib-0034] Silva, M. J. , Wong, L. Y. , Samandar, E. , Preau, J. L. , Calafat, A. M. , & Ye, X. (2017). Exposure to di‐2‐ethylhexyl terephthalate in a convenience sample of U.S. adults from 2000 to 2016. Archives of Toxicology, 91(10), 3293. 28314884 10.1007/s00204-017-1956-3 PMC5664933

[fsn31908-bib-0035] Sun, X. F. , Wang, S. G. , Zhang, X. M. , Chen, J. P. , Li, X. M. , Gao, B. Y. , & Ma, Y. (2009). Spectroscopic study of Zn2+ and Co2+ binding to extracellular polymeric substances (EPS) from aerobic granules. Journal of Colloid & Interface Science, 335(1), 11–17. 19419732 10.1016/j.jcis.2009.03.088

[fsn31908-bib-0036] Topcu, A. , Bulat, T. , Wishah, R. , & Boyac, I. H. (2010). Detoxification of aflatoxin B1 and patulin by *Enterococcus faecium* strains. International Journal of Food Microbiology, 139(3), 202–205. 20356644 10.1016/j.ijfoodmicro.2010.03.006

[fsn31908-bib-0037] Umpleby, R. J. , Baxter, S. C. , Chen, Y. , Shah, R. N. , & Shimizu, K. D. (2001). Characterization of molecularly imprinted polymers with the langmuir‐freundlich isotherm. Analytical Chemistry, 73(19), 4584–4591. 11605834 10.1021/ac0105686

[fsn31908-bib-0038] Wang, L. , Yue, T. , Yuan, Y. , Wang, Z. , Ye, M. , & Cai, R. (2015). A new insight into the adsorption mechanism of patulin by the heat‐inactive lactic acid bacteria cells. Food Control, 50, 104–110.

[fsn31908-bib-0039] Wicken, A. J. , & Knox, K. W. (1970). Studies on the group F antigen of lactobacilli: Isolation of a teichoic acid‐lipid complex from *Lactobacillus fermenti* NCTC 6991. Journal of General Microbiology, 60(3), 293–301. 5487616 10.1099/00221287-60-3-293

[fsn31908-bib-0040] Yin, K. , Lv, M. , Wang, Q. , Wu, Y. , Liao, C. , Zhang, W. , & Chen, L. (2016). Simultaneous bioremediation and biodetection of mercury ion through surface display of carboxylesterase E2 from Pseudomonas aeruginosa PA1. Water Research, 103, 383–390. 10.1016/j.watres.2016.07.053 27486950

[fsn31908-bib-0041] Zhang, D. , Liu, W. , Li, L. , Zhao, H. , Sun, H. , Meng, M. , Zhang, S. , & Shao, M. (2017). Key role of peptidoglycan on acrylamide binding by lactic acid bacteria. Food Science & Biotechnology, 26(1), 271–277. 30263538 10.1007/s10068-017-0036-z PMC6049493

[fsn31908-bib-0042] Zhao, H. , Zhou, F. , Qi, Y. , Dziugan, P. , Bai, F. , Walczak, P. , & Zhang, B. (2013). Screening of Lactobacillus strains for their ability to bind benzo(a)pyrene and the mechanism of the process. Food & Chemical Toxicology, 59(8), 67–71. 23747815 10.1016/j.fct.2013.05.040

[fsn31908-bib-0043] Zhao, L. , Wei, J. , Zhao, H. , Zhu, B. , & Zhang, B. (2018). Detoxification of cancerogenic compounds by lactic acid bacteria strains. Critical Reviews in Food Science and Nutrition, 58(16), 2727–2742. 10.1080/10408398.2017.1339665 29053003

[fsn31908-bib-0044] Zhao, L. , Zhao, H. , Shoukat, S. , Zhang, X. , & Zhang, B. (2017). Screening lactic acid bacteria strains with ability to bind di‐n‐butyl phthalate via Turbiscan technique. Antonie Van Leeuwenhoek, 110(6), 759–769. 28439679 10.1007/s10482-017-0846-2

[fsn31908-bib-0045] Zhu, Y. T. , Yang, C. X. , Luo, B. B. , Zhou, K. , & Liu, S. L. (2017). Efficiency of dairy strains of lactic acid bacteria to bind bisphenol a in phosphate buffer saline. Food Control, 73, 1203–1209.

